# *Chrysosplenetin* promotes osteoblastogenesis of bone marrow stromal cells via Wnt/β-catenin pathway and enhances osteogenesis in estrogen deficiency-induced bone loss

**DOI:** 10.1186/s13287-019-1375-x

**Published:** 2019-08-29

**Authors:** Guoju Hong, Xiaoming He, Yingshan Shen, Xiaojun Chen, Fang Yang, Peng Yang, Fengxiang Pang, Xiaorui Han, Wei He, Qiushi Wei

**Affiliations:** 1 Department of Surgery, The University of Alberta, Edmonton, Alberta Canada; 2 The National Key Discipline and the Orthopedic Laboratory, Guangzhou University of Chinese Medicine, Guangzhou, Guangdong People’s Republic of China; 3 School of Medicine, South China University of Technology, Guangzhou, Guangdong People’s Republic of China; 4 Department of Orthopedic, The First Affiliated Hospital of Guangzhou University of Chinese Medicine, Guangzhou, Guangdong People’s Republic of China; 5 Hip Preserving Ward, The First Affiliated Hospital of Guangzhou University of Chinese Medicine, No. 3 Orthopaedic Region, Guangzhou, Guangdong People’s Republic of China

**Keywords:** *Chrysosplenetin*, BMSC, Osteoblast, Wnt/β-catenin, DKK1, Noggin

## Abstract

**Background:**

*Chrysosplenetin* is an O-methylated flavonol compound isolated from the plant *Chamomilla recutita* and *Laggera pterodonta*. The aim of our research is to evaluate the function of *Chrysosplenetin* on osteogenesis of human-derived bone marrow stromal cells (hBMSCs) and inhibition of estrogen deficiency-induced osteoporosis via the Wnt/β-catenin signaling pathway.

**Method:**

hBMSCs are cultured and treated by *Chrysosplenetin* in the absence or presence of Wnt inhibitor dickkopf-related protein 1 (DKK1) or bone morphogenetic protein 2 (BMP2) antagonist Noggin. RT-qPCR is taken to identify the genetic expression of target genes of Wnt/β-catenin pathway and osteoblast-specific markers. The situation of β-catenin is measured by western blot and immunofluorescence staining. An ovariectomized (OVX) mouse model is set up to detect the bone loss suppression by injecting *Chrysosplenetin*. Micro-CT and histological assay are performed to evaluate the protection of bone matrix and osteoblast number. Serum markers related with osteogenesis are detected by ELISA.

**Results:**

In the present study, it is found that *Chrysosplenetin* time-dependently promoted proliferation and osteoblastogenesis of hBMSCs reaching its maximal effects at a concentration of 10 μM. The expressions of target genes of Wnt/β-catenin pathway and osteoblast-specific marker genes are enhanced by *Chrysosplenetin* treatment. Furthermore, the phosphorylation of β-catenin is decreased, and nuclear translocation of β-catenin is promoted by *Chrysosplenetin*. Osteogenesis effects mentioned above are founded to be blocked by DKK1 or BMP2 antagonist Noggin. *In vivo* study reveals that *Chrysosplenetin* prevents estrogen deficiency-induced bone loss in OVX mice detected by Micro-CT, histological analysis, and ELISA*.*

**Conclusions:**

Our study demonstrates that *Chrysosplenetin* improves osteoblastogenesis of hBMSCs and osteogenesis in estrogen deficiency-induced bone loss by regulating Wnt/β-catenin pathway.

**Electronic supplementary material:**

The online version of this article (10.1186/s13287-019-1375-x) contains supplementary material, which is available to authorized users.

## Introduction

Postmenopausal osteoporosis (PO) is characterized by bone mineral reduced and architectural deterioration in the skeletal system due to deficiency of estrogen [1, 2]. Basically, PO is one kind of dynamic pathologic condition resulting from an imbalance between osteoclastic resorption and osteoblastic formation. The suppressed viability and less differentiation of osteoblast mainly result from reduced proliferation potential of bone marrow stromal cells (BMSCs), especially in aged menopausal women [3]. If women suffer from insufficient estrogen support, BMSCs will turn less into osteoblast and conduct osteoporosis [4]. Hence, keeping the activity of BMSCs is crucial to maintain the amount and potential of osteoblast in bone loss.

It is highlighted that Wnt/β-catenin signaling pathway plays a great role in the bone homeostasis [5, 6]. Wnt is a family member of the secreted lipid-modified signaling glycoproteins and is triggered to combine with its receptors mainly by palmitoleoylation [7]. To be specific, Wnt ligand combines with Frizzled (Fz) and low-density lipoprotein receptor-related protein 5/6 (LRP5/6). The protein complex activates the scaffolding protein Dishevelled (Dvl) and motivates the phosphorylation of LRP5/6, then leading to the recruitment of Axin. The above biochemical actions finally attenuate the phosphorylation β-catenin and enhance the accumulation of β-catenin in nucleus of cells. Canonical Wnt/β-catenin has an essential effect on BMSC commitment stage and enhance tissue ossification [8]. It was reported that the absence of Wnt16 in knockout mice resulted in decrease of canonical markers (β-catenin, Axin) and less bone mass formation [9]. Hence, targeting Wnt/β-catenin signaling pathway is a promising strategy to promote insufficient bone resorption, thereby alleviating bone loss.

**Fig. 1:**
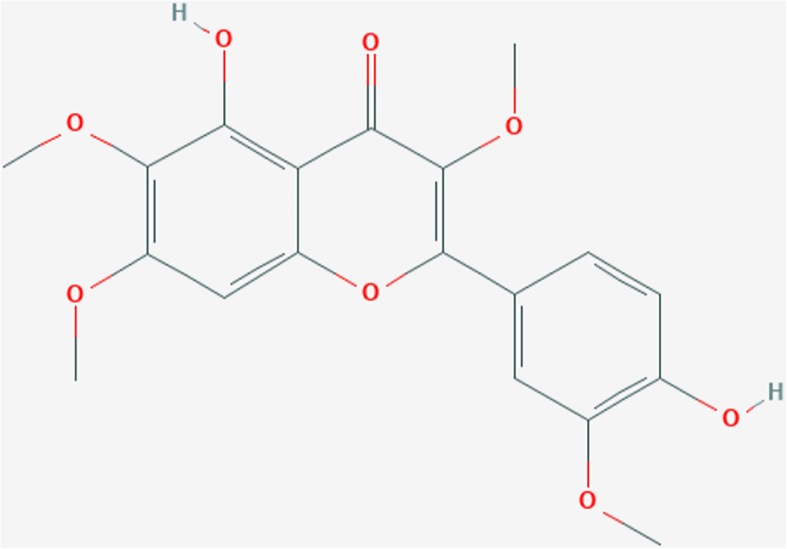
Chemical structure of *Chrysosplenetin* (cited from PubChem substance CID 5281608)

*Chrysosplenetin* is an active O-methylated flavonol extracted from *Chamomilla recutita* and *Laggera pterodonta* (Fig. 1). Previous researches proposed multiple O-methylated flavonols or isoflavones were beneficial to osteogenesis through activating the viability of osteoblast, such as Syringetin [10] and Tectorigenin [11]. Hence, *Chrysosplenetin* is a promising compound for the induction of osteoblast formation and bone formation. *Chrysosplenetin* has been utilized as one kind of natural compound in treating cancer [12] and anti-enterovirus infection [13]. It is reported that *Chrysosplenetin* had antitumor properties for breast cancer. Cytotoxic activity against breast cancer cells is detected after the compound’s treatment and leads to its apoptosis by regulating microtubule depolymerization [12]. *Chrysosplenetin* also contributes greatly to enhancing the activity of acetylcholinesterase (AChE) for anti-inflammation and neuro disease [14]. However, thus far, its effect and molecular mechanism on bone homeostasis still remained unknown. Therefore, we try to investigate the effect of *Chrysosplenetin* on osteoblastogenesis of BMSCs.

In our study, we innovatively identify the effects of *Chrysosplenetin* on osteoblastogenesis of hBMSCs *in vitro*. It is indicated that *Chrysosplenetin* promotes osteoblastic differentiation of hBMSCs and increases canonical Wnt/β-catenin signaling pathway. Furthermore, an ovariectomized (OVX) mouse model was set up to investigate the physiological efficiency of *Chrysosplenetin* on PO *in vivo*. The result suggests that estrogen deficiency-induced bone loss is significantly inhibited by *Chrysosplenetin* treatment. Our study has revealed that *Chrysosplenetin* may improve osteoblastogenesis of BMSCs via Wnt/β-catenin signaling pathway and prevent estrogen deficiency-induced bone loss.

## Materials and methods

### Materials and reagents

*Chrysosplenetin* (purify ≥ 98%) was obtained from the TransMIT Project Division for Plant Metabolites and Chemicals (Gießen, Germany) and dissolved in dimethyl sulfoxide (DMSO) purchased from Sigma-Aldrich (St. Louis, MO, USA). It was firstly dissolved as a primary concentration of 10 mM and then diluted to final concentrations as required in the culture medium. Low-glucose Dulbecco minimum essential medium (LG-DMEM), penicillin-streptomycin, and fetal bovine serum (FBS) were ordered from Thermo Fisher Scientific (Waltham, MA, USA). Human Dickkopf-related protein 1 (DKK1) and Noggin recombinant protein were obtained from Sigma-Aldrich (St. Louis, MO, USA) and dissolved in bovine serum albumin (BSA) (Thermo Fisher Scientific, Waltham, MA, USA). β-Catenin antibody, phosphorylation-β-catenin (p- β-catenin), and β-actin antibody were purchased from Cell Signaling Technology (Whitby, Ontario, USA). All antibodies were used at the concentrations recommended by the supplier at 1:1000. Second antibodies were purchased from Thermo Fisher Scientific (Waltham, MA, USA). Mineral deposition of osteoblast was detected using the Alizarin Red Staining Kit (Sigma-Aldrich, St. Louis, MO, USA). Alkaline Phosphatase (ALP) Activity Fluorometric Assay Kit was purchased from Abcam (Cambridge, MA, USA). ELISA kit of *Ot/Bgp*, *Balp*, and *Ct* were obtained from R&D Company (Minneapolis, MN, USA). Human bone marrow stromal cells (hBMSCs) were obtained from Cyagen Biosciences Inc. (Guangzhou, China).

### hBMSCs culture and surface antigen identification

hBMSC suspension was seeded into a culture flask and cultured in a completed DMEM medium (LG-DMEM, 10% FBS, 100 U/ml penicillin and 100 mg/mL streptomycin). The monolayer culture of hBMSCs was kept in an incubator with a condition as 5% CO^2^ at 37 °C. The medium was replaced for every 3 days until the flask was confluence and filled with cells. Then, hBMSCs were digested and passaged to the next generation for up to three times. The hBMSC suspension for each passage was grouped. The cell-surface antigen (CD73, CD14, CD44, CD45, CD106, CD105, CD29, CD11b, CD34, Human Leukocyte Antigen-DR isotype (HLA-DR) and correspondence isotype control) of the hBMSCs was identified. After that, hBMSCs were washed twice with phosphate-buffered saline (PBS) and fixed in 1.0% paraformaldehyde (PFA). Flow cytometry was undertaken for the evaluation of surface antigen. Subsequently, hBMSCs from 2 and 3 passages were used for the following experiment in our test.

### hBMSC proliferation assay

hBMSC suspension were plated in 96-well plates (1 × 10^4^ cells/well) for overnight incubation. *Chrysosplenetin* (5, 10, and 20 μM) was then added to cells in the presence of osteogenic induction medium (OIM, containing 0.1 mM dexamethasone, 50 mM l-ascorbic acid-2-phosphate, and 10 mM b-glycerophosphate). Plates with cells were incubated at 37 °C for 1, 2, 3, 7, and 14 days. MTT assay was measured to identify the effect of *Chrysosplenetin* on the proliferation of hBMSCs. 3-(4,5-Dimethylthiazol- 2-yl)-2,5-diphenyltetrazolium bromide was added into each well for further 3 h. Then, the formazan crystals were dissolved in DMSO. The absorbance was evaluated at 490-nm wavelength to assess the viability of hBMSCs.

### Strategies of compound testing

In our test, there are two strategies for the following evaluation. First, in order to evaluate the effect as well as optimal concentration of *Chrysosplenetin* for osteogenic differentiation of hBMSCs, cells were added by OIM supplemented with *Chrysosplenetin* at several concentrations (5, 10 and 20 μM) for 14 days. Second, to determine the effects of *Chrysosplenetin*, Noggin, and DKK1 on the osteogenic differentiation of hBMSCs, hBMSCs with OIM were treated by *Chrysosplenetin* at an optimal concentration in the presence or absence of Noggin (10 μg/ml) or DKK1 (0.5 μg/ml).

### Alizarin red staining and ALP activity assay

hBMSCs were seeded and cultured in the 6-well plates at a density of 1 × 10^6^ per well for osteoblastogenesis. After osteogenic induction in strategy one, alizarin red staining and ALP activity assay were performed to identify bone mineral of cells according to the manufacturers’ introduction. Specifically, cells were cultured in varying concentrations of *Chrysosplenetin* for 14 days and fixed with 4% PFA for 30 min, followed by PBS washing. Alizarin red staining solution was added into the wells for 5 min. The cells were viewed using a 450 fluorescent inverted phase-contrast microscope (Nikon Corporation, Tokyo, Japan).

As for ALP activity assay, hBMSCs were modulated same as what in alizarin red staining test. Then, cell lysates were collected in a test tube with alkaline solution and subjected to ALP activity analysis by a fluorometric detection kit. The absorbance set up as 450 nm was measured using a microplate reader (ELx800, BioTek, Winooski, VT, USA).

### RNA extraction and real-time polymerase chain reaction (real-time PCR)

For real-time PCR, hBMSCs were seeded in 6-well plates at a density of 1 × 10^6^ cells per well. In strategy one, the mRNAs of osteoblastic genes including Runt Related Transcription Factor 2 (*RUNX2)*, Osteocalcin (*BGLAP*), β-catenin (*CTNNB1*), and Bone Morphogenetic Protein 2 (*BMP2*) were detected. In strategy two, the mRNAs of osteogenic genes, including *RUNX2*, Distal-less Homeobox 5 (*DLX5*), Osteopontin (*SPP1*), Collagen type I (*COL1*), *BGLAP*, and *BMP2*, and Wnt/β-catenin target genes, including *CTNNB1*, Transcription Factor 7 (*TCF7*), Lymphoid Enhancer Binding Factor 1 (*LEF1*), MYC (*C-MYC*), cyclin D (*CCND1*), and c-JUN (*JUN*), were identified for analysis. PCR reactions using specific primers of the genes were seen in the Additional file 1.

hBMSCs after osteogenic induction mentioned above were lysed. Then, the total RNA was isolated from the hBMSCs by adding Trizol reagent (Life Technologies, Sydney, Australia) in accordance with the product instruction. Concentration of RNA was determined by Thermo Scientific Microplate Reader (Thermo, USA), and single-stranded cDNA was reverse transcribed. qPCR reactions were performed in a ViiA 7 Real-time PCR system (Thermo Fisher Scientific, Waltham, MA, USA). Polymerase chain reaction amplification of specific sequences was cycled in the following condition: 94 °C for 5 min, followed by 30 cycles of 94 °C (40 s), 60 °C, (40 s), and 72 °C (40 s), and a final dissociation of 30 s at 72 °C. Data was showed as Ct (2^−∆∆CT^) and normalized against *GAPDH.* Reaction products were separated using agarose gel electrophoresis and visualized on an Image-quant LAS 4000 (GE Healthcare, Silverwater, Australia).

### Western blot assay

Fresh hBMSCs were seeded into 12-well plates (5 × 10^5^ cells/well) and pre-treated with OIM for 3, 7 and 14 days. hBMSCs in the *Chrysosplenetin* test group were treated with or without Noggin or DKK1*.* After that, cells were lysed with RIPA Lysis buffer (Thermo Fisher Scientific, Waltham, MA, USA) for protein extraction and pelleted at 14,000×*g* for 5 min. Proteins were identified by Bradford Protein Assay Kit (Thermo Scientific, MA, USA). Samples then were separated using 10% SDS-polyacrylamide gel electrophoresis (SDS-PAGE) loading buffer electrophoresis (Sigma-Aldrich, St. Louis, MO, USA) and transferred onto nitrocellulose membranes (GE Healthcare, Silverwater, Australia). The membrane was blocked in 5% skim milk diluted in 1× TBS-Tween (TBST, containing 10 mM Tris-HCl (pH 7.6), 150 mM NaCl, 0.05% Tween-20) for 1 h at room temperature, then probed with specific antibodies, including p-β-catenin and β-catenin antibodies (diluted as 1:1000) at 4 °C for overnight in a shaking manner. The membrane was washed in TBST and incubated in appropriate horseradish peroxidase-conjugated secondary antibodies (1:5000). The membrane was then developed using the ECL reagents (Amersham Pharmacia Biotech, Sydney, Australia). Band intensity was normalized against *β*-actin. Finally, an Image quant LAS 4000 (GE Healthcare, Silverwater, Australia) was used to figure out the membranes.

### Immunofluorescence staining

hBMSCs that undergone modulation of strategy one were fixed in 4% paraformaldehyde in PBS for 15 min, rinsed in 0.25% Triton X-100 in PBS, and subsequently blocked in 1% BSA in poly butylene succinate-co-butylene terephthalate (PBST, 0.05% Tween-20 in PBS) for 30 min. After three washes of PBS with 5 min for each time, cells were incubated for 1 h with primary anti-β-catenin antibodies at 1:100 dilution at room temperature. After the first-round incubation and three washes of PBS again, cells were incubated for 30 min with fluorescein isothiocyanate (FITC)-linked goat anti-rabbit IgG conjugated at 1:100 dilution. Finally, cell samples were washed for 40-6-Diamidino-2-phenylindole (DAPI, 1:1000 dilution, Thermo Fisher Scientific, Waltham, MA, USA) staining to highlight nuclei of the cells. Then, the cellular samples were observed by using a confocal microscope (Fluoview 300, Olympus, Tokyo, Japan), and captured images were acquired using the Image-Pro Plus 6.0 software (Media Cybernetics, Rockville, MD, USA).

### OVX mouse model

Totally, 18 specific pathogen-free C57BL/6 J female mice (7 weeks of age) were randomly divided into three groups and six mice for per group (sham group, OVX group, and test group (3 mg/kg *Chrysosplenetin* injection). The mice were kept individually in cages with standard chow and water support and a half-day light/dark cycle. The OVX group and test group were anesthetized and undertaken an ovariectomy inducing estrogen deficiency, whereas the sham operation was performed in the sham group as a normal control. Each ovary in the OVX group and test group was completely removed with its capsule and part of the oviduct. All of groups had 1 week for recovery and incision healing. After that, mice in the test group were given an intraperitoneal injection of *Chrysosplenetin* at 3 mg/kg for every 2 days, instead of 1% DMSO in the sham and OVX groups. At 6 weeks post-treatment, the mice were euthanized. The femur was removed and fixed in 4% PFA for 24 h. The protocols involving animal model were approved by Guangzhou University of Chinese Medicine Institutional Animal Ethics Committee.

### Micro-CT and bone histomorphometric analyses

After the model was established, the femur without excess soft tissue was isolated from each group and fixed in 10% neutral-buffered formalin. The femur was then washed and put in PBS for soaking. The prepared bone was transferred to a tube of the Skyscan 1176 Micro-CT equipment (Skyscan, Aartselaar, Belgium) for scanning. The parameters of scanning setting were as follows: 70 kV, 200uA, 10-μm Al filter, 300-ms exposure, pixel size 8.89 μm, 2 frame averaging, and 0.4-degree rotation step through 180°. A resolution as high as 9 μm was obtained in scanning CT images, and 3D reconstruction was established. We determined the proximal femur as our region of interest (ROI), to be specific, volume starting 0.5 mm from the bottom of the growth plate for a 1-mm distance. The several parameters of trabecular bone within this volume including volume/total volume (BV/TV), trabecular number (Tb.N), trabecular thickness (Tb.Th), and trabecular separation (Tb.Sp) were manually identified by a constant threshold.

### Histological assay and enzyme-linked immunosorbent assay (ELISA)

By following the micro-CT analysis, bone samples were fixed with 4% PFA and then decalcify later. Samples were cut and prepared as the sequential 5-mm-thick sections stained using hematoxylin and eosin (H&E). As for immunohistochemistry, the sections were de-paraffinized and rehydrated and incubated with primary antibody against (1:600) overnight at 4 °C. For detection, sections were incubated with HRP-conjugated secondary antibody for 60 min, followed by the addition of liquid DAB substrate (Thermo Fisher Scientific, Waltham, MA, USA). Sections were counterstained with hematoxylin and were dehydrated and mounted with fast-drying mounting media (United Biosciences, Australia). Sections were scanned using Aperio Scanscope (Leica Biosystem, Buffalo Grove, IL, USA), and bone histomorphometric analyses were performed using BIOQUANT OSTEO software (Bioquant Image Analysis Corporation, Nashville, TN, USA).

Serum was isolated from the abdominal aorta of mice in each group, and the level of Osteocalcin/Bone Gla Protein (*Ot/Bgp*), Bone Alkaline Phosphatase (*Balp*), and Calcitonin (*Ct*) was assessed using ELISA kit according to the manufacturers’ introduction.

### Statistical analysis

All original experimental values are reported as the mean ± standard deviation (SD) of the values obtained from three repeated experiments. All the experiments were repeated at least three times and presented as the average of triplicate independent experiments or the data from a representative experiment. Statistical significance was determined by Student’s *t* test. A *p* < 0.05 was considered statistically significant.

## Results

### Characteristic and phenotypic identification of hBMSCs

**Fig. 2:**
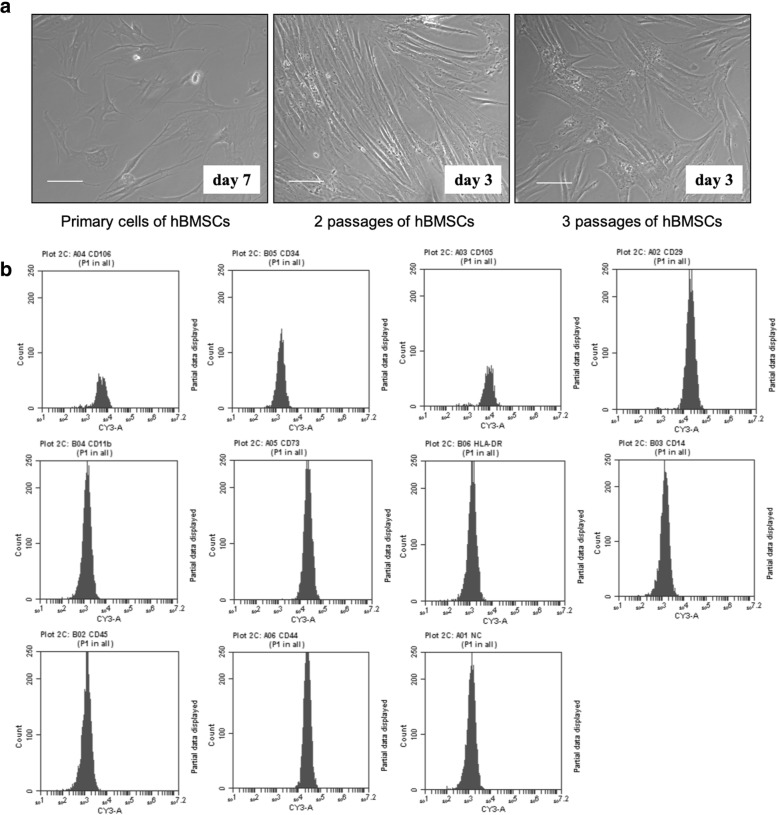
Differentiation and identification of human mesenchymal stem cells (hBMSCs). **a** Representative images demonstrate that primary hBMSCs were incubated and reached 90% confluence at day 7 while cells at passages 1 and 2 at day 3. Scale bar = 100 μm. **b** Flow cytometry was taken to measure the levels of the specific biomarkers CD73, CD14, CD44, CD45, CD106, CD105, CD29, CD11b, CD34, and Human Leukocyte Antigen-DR isotype (HLA-DR) in hBMSCs

To identify the characteristic and phenotypic of hBMSCs, primary cell culture and surface marker detection were performed. hBMSCs were expanded in primary culture and passaged for 3 to 5 times. In primary phase, attached hBMSCs obtained a 90% confluence at days 7. hBMSCs at passages 1 and 2 reached 90% confluence at day 3 (Fig. 2a). Then, surface markers were evaluated by flow cytometry. As shown in Fig. 2b, the outcome data demonstrated that there were no specific surface markers. The results of cells were positive for CD73, CD106, CD105, CD29, and CD44, as 99.9%, 76.9%, 92.5%, 99.0%, and 100.0%, whereas negative for CD14, CD45, CD34, CD11b, and HLA-DR as 0.3%, 0.4%, 0.8%, 0.3%, and 0.4%.

### Chrysosplenetin enhanced proliferation and osteoblastogenesis of hBMSCs

**Fig. 3:**
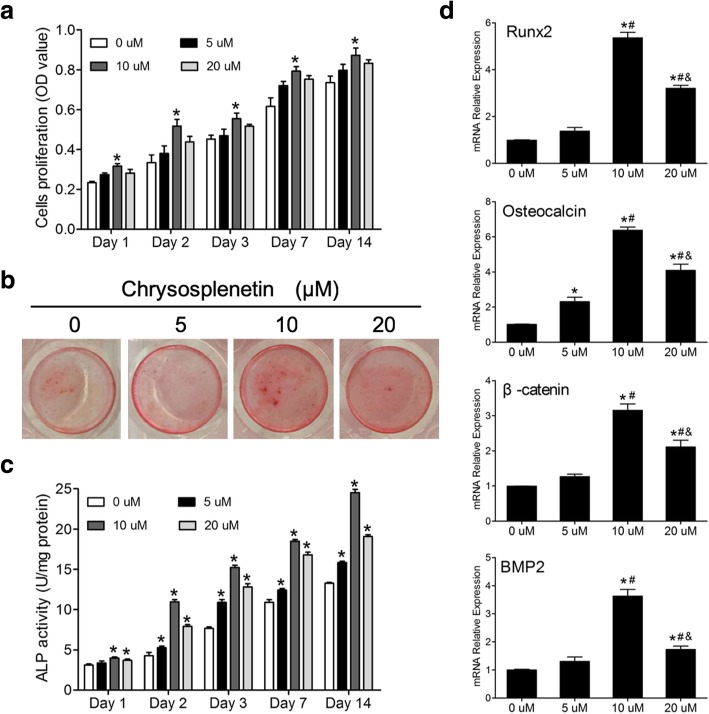
*Chrysosplenetin* improves proliferation and osteogenic differentiation of hBMSCs. **a** MTT assay was undertaken to measure the proliferation of hBMSCs. hBMSCs were cultured with osteogenic induction medium (OIM) and various concentrations of *Chrysosplenetin* for 1, 2, 3, 7, and 14 days (*p < 0.05 relative to OIM-stimulated controls). **b** Representative images demonstrated that mineral deposit of hBMSCs treated by *Chrysosplenetin* (10 μM) was tested by Alizarin red. **c** Analysis of alkaline phosphatase (ALP) activity in hBMSCs treated with *Chrysosplenetin* (0, 5, 10, 20 μM) (*p < 0.05 relative to OIM-stimulated controls at the same time point). **d** RT-pPCR was used to evaluate the expression of osteogenic genes in hBMSCs cells planted with OIM in the presence or absence of *Chrysosplenetin* at 5, 10, and 20 μM. Gene expression was normalized to *GAPDH* (Runt Related Transcription Factor 2 (*RUNX2)*; Osteocalcin (*BGLAP*); β-catenin (*CTNNB1*); Bone Morphogenetic Protein 2 (*BMP2*), *p < 0.05 relative to OIM-stimulated controls; ^#^*p* < 0.01 relative to 10 μM group; ^&^*p* < 0.01 relative to 30 μM group)

To address the direct effect of *Chrysosplenetin* on proliferation of hBMSCs, cells were cultured in basal medium according to strategy one and MTT assay was performed. The result showed that the proliferation of hBMSCs was promoted in the presence of *Chrysosplenetin* at 5, 10, and 20 μM, compared to the untreated control group. The maximal positive effect was achieved by an optimal concentration as 10 μM (Fig. 3a). As shown in Fig. 3b, alizarin red staining revealed significant difference between the nodule formation in the control group and *Chrysosplenetin* treatment groups. Ten micromolar *Chrysosplenetin* can induce more nodular formation. And a non-dose-dependent increase of ALP activity was also observed by *Chrysosplenetin* (Fig. 3c). There were consistent results of these two tests that 10 μM of *Chrysosplenetin* enable to promote osteogenesis differentiation of hBMSCs to a maximal level.

### Chrysosplenetin increased osteogenic gene expression

Next, we use real-time PCR to estimate the effect of *Chrysosplenetin* on osteogenic gene expression levels during differentiation of hBMSCs. Consistent with osteoblast formation and activity assays, gene expression of the osteoblastic marker genes *RUNX2*, *BGLAP*, *CTNNB1*, and *BMP2* increased significantly in a non-dose-dependent manner by *Chrysosplenetin* (0, 5, 10, and 20 μM) at day 14 of culture (Fig. 3d).

### Chrysosplenetin activated target genes of Wnt/β-catenin pathway

**Fig. 4:**
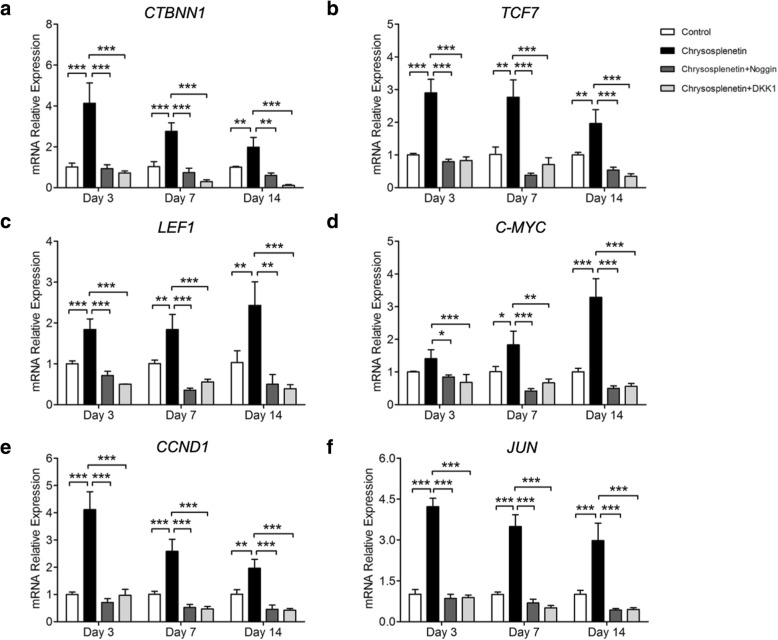
*Chrysosplenetin* increases Wnt/β-catenin pathway target gene expression in hBMSCs. RT-pPCR was performed to assess the expressions of target genes of Wnt/β-catenin pathway. hBMSCs cells were seeded with OIM and *Chrysosplenetin* (10 μM) in the presence or absence of Noggin (10 μg/ml) or DKK1 (0.5 μg/ml) for 3, 7, and 14 days. Gene expression was normalized to *GAPDH*; **a** *CTNNB1*; **b** Transcription Factor 7 (*TCF7*); **c** Lymphoid Enhancer Binding Factor 1 *(LEF1)*; **d** MYC (*C-MYC*); **e** cyclin D (*CCND1*); **f** c-JUN (*JUN*) (*p < 0.05, **p < 0.01, ***p < 0.001 relative to *Chrysosplenetin* treating group)

Wnt/β-catenin pathway plays an important role in the enhancement of hBMSC differentiation. Here qRT-PCR was taken to measure the expression of *CTNNB1*, *TCF7*, *LEF1*, *CCND1*, *JUN*, and *c-MYC* by treating with *Chrysosplenetin* in the presence or absence of Noggin and DKK1. It was suggested, compared with control group, *Chrysosplenetin* elevated the potential of OIM and markedly increased the mRNA levels of *CTNNB1*, *TCF7*, *LEF1*, *CCND1*, *JUN*, and *c-MYC* at various time points ranging from 3 to 14 days at a relative time dependence. By pretreating with Noggin and DKK1 into hBMSCs, expressions of target genes were greatly downregulated to a level lower than that of the control group (Fig. 4a–f).

### Chrysosplenetin inhibits downstream osteogenic genes

**Fig. 5:**
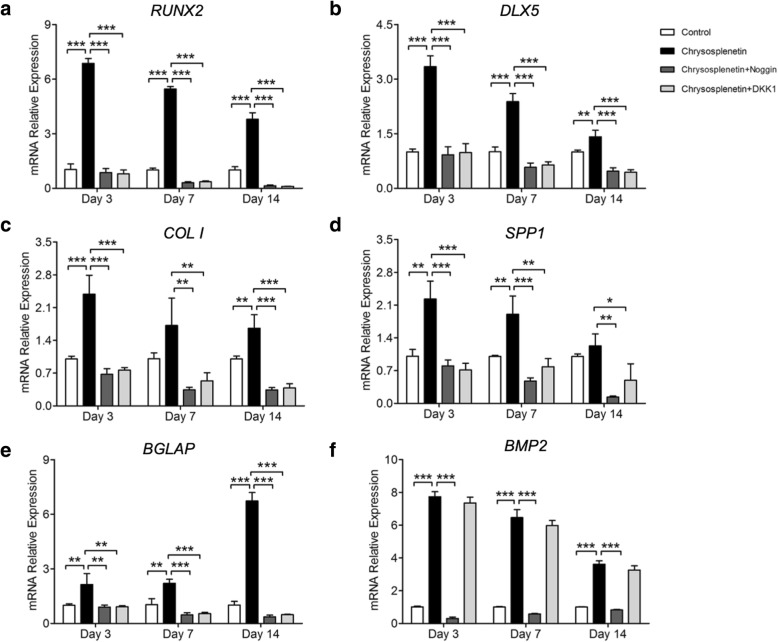
*Chrysosplenetin* promotes osteogenic gene expression in hBMSCs. RT-pPCR was utilized to evaluate the expression of osteogenic genes. hBMSCs cells were planted and added with OIM and *Chrysosplenetin* (10 μM) in the presence or absence of Noggin (10 μg/ml) or DKK1 (0.5 μg/ml) for 3, 7, and 14 days. Gene expression was normalized to *GAPDH*; **a** *RUNX2*; **b** Distal-less Homeobox 5 (*DLX5*); **c** Osteopontin (*SPP1*); **d** Collagen type I (*COL1*); **e** *BGLAP*; **f** BMP2 (*p < 0.05, **p < 0.01, ***p < 0.001 relative to *Chrysosplenetin* treating group)

Wnt/β-catenin signaling promotes the osteoblastic formation of hBMSCs via several downstream osteogenic genes like *RUNX2*, *DLX5*, *SPP1*, *COL1*, *BGLAP*, and *BMP2*. It was indicated that *Chrysosplenetin* at 10 μM upregulated the expression of β-catenin and TCF7 on 3 and 7 days compared with the control group. Moreover, it significantly increased the expression of *RUNX2*, *DLX5*, *SPP1*, *COL1*, and *BGLAP* on 3, 7, and 14 days, which were significantly reversed by the pretreatment with Noggin and DKK1 (Fig. 5a–e). Particularly, the elevated expression of *BMP2* mRNA which was promoted by *Chrysosplenetin* was greatly suppressed by Noggin, but no antagonistic effect of DKK1 was observed during the differentiation (Fig. 5f).

### Chrysosplenetin promotes phosphorylation of β-catenin

**Fig. 6:**
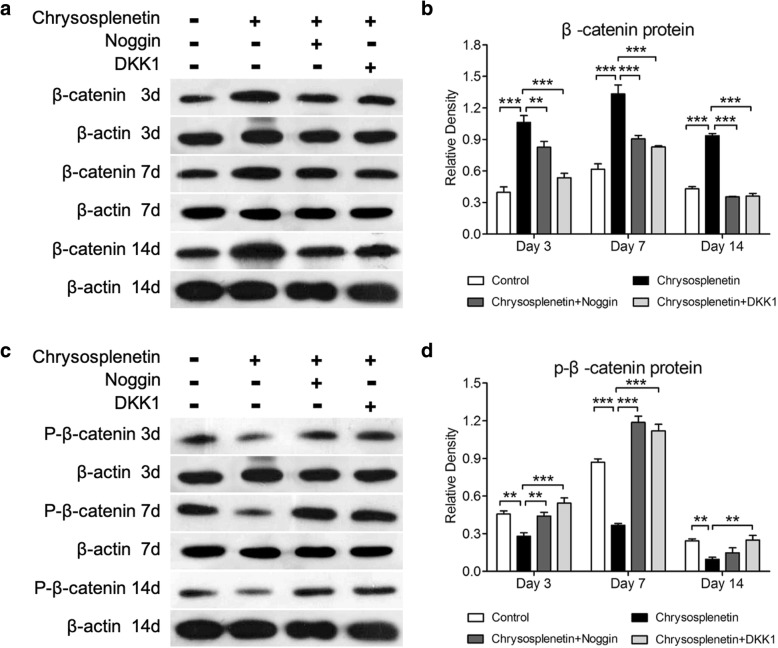
The effect of *Chrysosplenetin* on total and phosphorylation of β-catenin. Protein lysates from OIM-induced hBMSCs were pre-treated with *Chrysosplenetin* (10 μM), which were added with Noggin (10 μg/ml) or DKK1 (0.5 μg/ml) at days 3, 7, and 14. The protein expressions of β-catenin (**a**) and p-β-catenin (**b**) were tested by Western blot assay with specific antibodies. Relative results were expressed by the ratio of the amount of β-catenin/*β*-actin (**c**) and p-β-catenin/*β*-actin (**d**) determined by Image J (*p < 0.05, **p < 0.01, ***p < 0.001 relative to *Chrysosplenetin*-treated group)

In order to determine the impact of *Chrysosplenetin* on the phosphorylation of β-catenin in the Wnt/β-catenin signaling pathway, the protein expressions of β-catenin and p-β-catenin were detected by western blot assay at different time points. The result demonstrated that *Chrysosplenetin* increased the expression of β-catenin through the culture compared to control group and reach its peak of expression at 7 days. This effect was greatly decompressed by Noggin and DKK1 respectively as expected (Fig. 6a, b). p-β-catenin expression was in contrast to that of β-catenin completely. The protein expression of p-*β*-catenin in the control group increase from 3 to 7 days and decline at 14 days. By comparing with the control group, the level of p-β-catenin was significantly suppressed by *Chrysosplenetin*. However, these effects were reversed during the culture process by adding Noggin and DKK1 (Fig. 6c, d).

### Chrysosplenetin actives β-catenin nuclear translocation

**Fig. 7:**
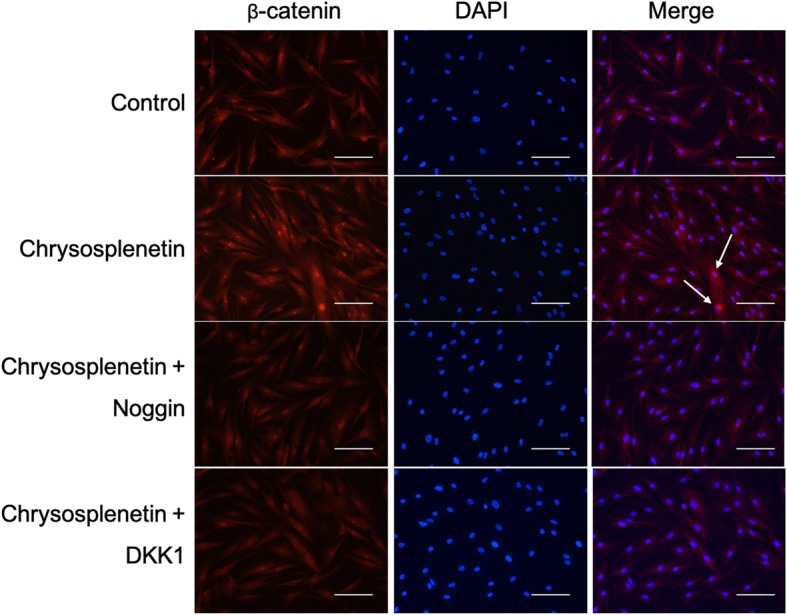
*Chrysosplenetin* activates the nuclear translocation of β-catenin in hBMSCs. Scale bar (white line): 100μM

When considering the Wnt/β-catenin pathway, the successful nucleus translocation of β-catenin is directly associated with the activation of the signaling pathway. In order to further investigate the potential effect of *Chrysosplenetin* on the induction of β-catenin translocation into the nucleus, immunolabeling and fluorescence microscopy of β-catenin were undertaken. The result demonstrated that less expression of β-catenin (stained with red color) in the nucleus of the hBMSCs (stained with blue color) was observed in the control group. By adding *Chrysosplenetin* (10 μM) into hBMSCs, obviously enhanced translocation of β-catenin was noticed in the nucleus. However, pretreatment with Noggin and DKK1 enable to block the *Chrysosplenetin* significantly by reducing the nuclear translocation with low level of β-catenin in merged images (Fig. 7).

### Chrysosplenetin suppressed estrogen deficiency bone loss in OVX mice

**Fig. 8:**
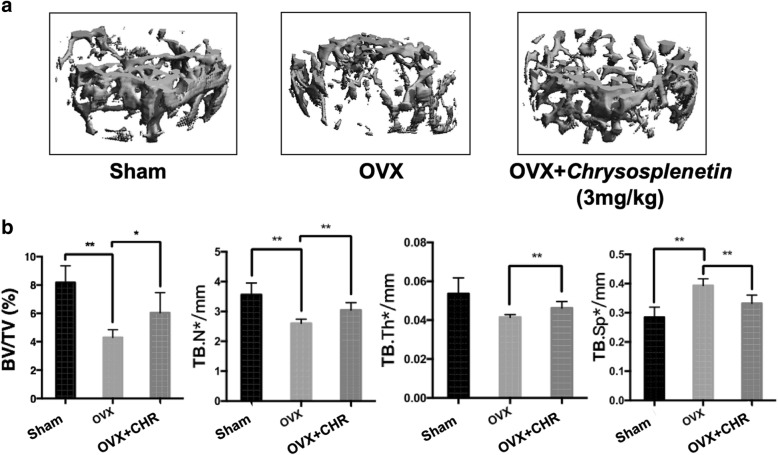
*Chrysosplenetin* inhibit ovariectomy estrogen deficiency-induced bone resorption. **a** Representative 3D reconstruction image and micro-CT analysis of trabecular bone microarchitecture from the femur of sham mice, OVX mice, and test group treated with *Chrysosplenetin* at 3 mg/kg. The result shows the potential protective effect of *Chrysosplenetin* in OVX-induced osteoporosis. **b** Quantitative analyses of bone volume/total volume (BV/TV), trabecular number (Tb.N*), trabecular thickness (Tb.Th*), and trabecular separation (Tb.Sp*) (*n* = 6) (CHR *Chrysosplenetin*; *p < 0.05, **p < 0.01 relative to OVX untreated controls)

In order to evaluate the role of *Chrysosplenetin* on estrogen deficiency bone loss, mice were given OVX surgery and treated with either *Chrysosplenetin* (3 mg/kg) or DMSO only for control. There was no adverse event discovered during the whole procedure. In micro-CT assessment, the result revealed that *Chrysosplenetin* significantly reduced the bone loss associated with ovariectomy, as shown by upregulations of BV/TV, Tb.N, and Tb.Th in test mice. Trabecular separation (Tb. Sp) was prevented in the test group when compared with the OVX control group (Fig. 8a, b). Taken together, the results suggested that *Chrysosplenetin* protected against estrogen deficiency bone loss.

**Fig. 9:**
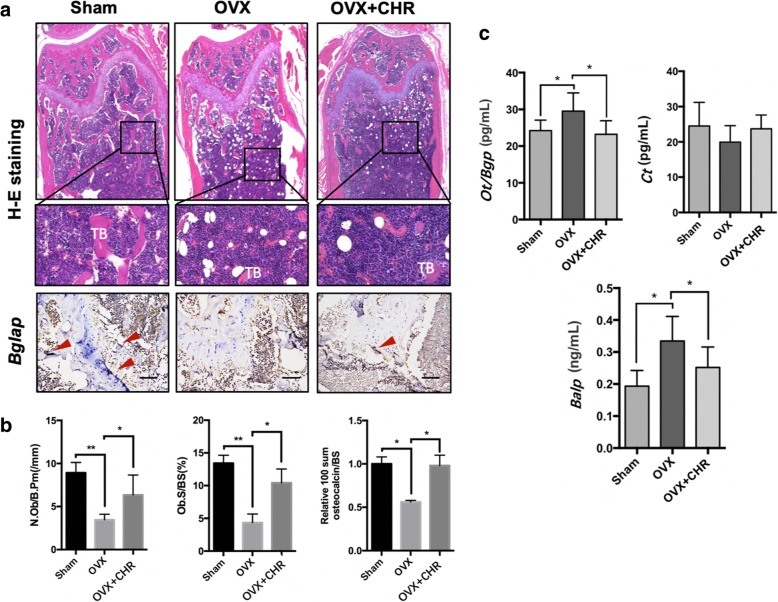
*Chrysosplenetin* prevents OVX mouse model from bone loss via promoting osteoblast activity. **a** Representative images of decalcified bone stained with H&E and immunochemically stained with *Bglap* on sham mice, OVX mice, and test group treated with 3 mg/kg *Chrysosplenetin*. Red arrow indicates high expression of *Bglap*; scale bar: 100 μm; TB, Trabecula. **b** Quantitative analyses of N.Ob/B.Pm (/mm) (osteoblasts number/bone perimeter) and Ob.S/BS(%) (osteoblast surfaces/bone surface) of mice. Relative *Bgalp* expression in mouse femurs (*n* = 6). **c** Quantitative analyses of Osteocalcin/Bone Gla Protein (*Ot/Bgp*), Bone Alkaline Phosphatase (*Balp*), and Calcitonin (*Ct*) in the serum of mice (*n* = 6) (*p < 0.05 and **p < 0.01 relative to OVX untreated controls)

The histomorphometric analysis was used to evaluate the function of *Chrysosplenetin* on estrogen deficiency bone loss. There was an increase in BV/TV in the test group consistent with that of micro-CT (Fig. 9a, b). Our results also showed that there was a significant difference between *Chrysosplenetin* treatment and the OVX group in N.Ob/B.Pm(/mm) (osteoblasts number/bone perimeter) and Ob.S/BS(%) (osteoblast surfaces/bone surface) as well as *Bgalp* expression in bone tissue (Fig. 9a, b). Here we concluded that *Chrysosplenetin* protected from estrogen deficiency bone loss by promoting osteoblast number and activity. In ELISA, osteoblastic markers in serum of mice, including *Ot/Bgp* were evaluated in OVX mouse model and suppressed in addition of *Chrysosplenetin* injection, and *Balp* vice versa. Expression of *Ct* in serum was in opposite of the former two markers (Fig. 9c).

## Discussion

*Chrysosplenetin* is a novel natural compound produced by the *Chamomilla recutita* and *Laggera pterodonta*, which is famous in treating inflammation of multiple systems in ancient Asia, especially in China, Korea, and Japan. Recently, *Chrysosplenetin* has been found to suppress breast cancer cells [12], inflammation [14], intestinal disease [13, 15], and so on. In addition, *Chrysosplenetin* is able to reverse the pharmacokinetic disadvantages of *Artemisinin* greatly. *Artemisinin* has one semisynthetic derivative called dihydroartemisinin, and it is tested to be effective in the treatment of bone loss [16, 17]. Hence, we hypothesized that *Chrysosplenetin* might also have its unrevealed effect on deficiency of osteogenesis. In this study, we explored the mechanism of *Chrysosplenetin* on osteoblast formation of BMSCs and activity, and through an OVX-induced bone loss mouse model.

**Fig. 10:**
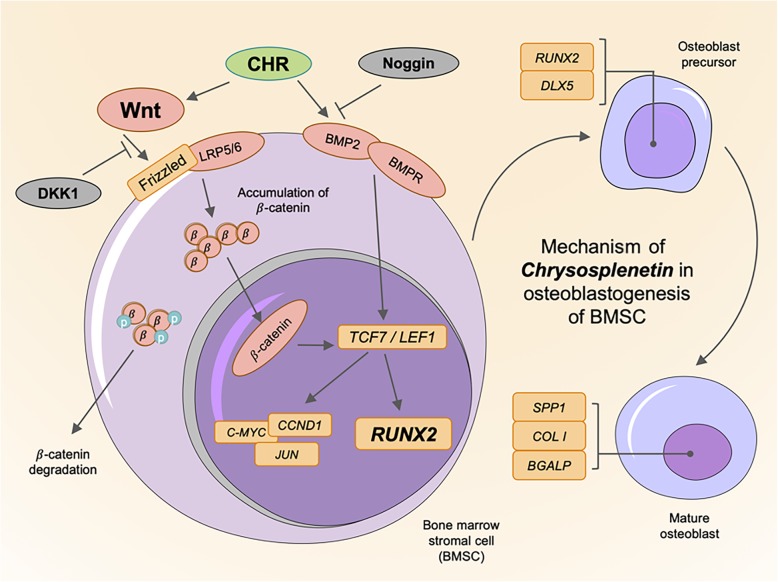
Hypothetical scheme helps in understanding the mechanism of *Chrysosplenetin* in Wnt/β-catenin pathway. (DKK1, Dickkopf-related protein 1; CHR, Chrysosplenetin; RUNX2, Runt Related Transcription Factor 2; BGLAP, Osteocalcin; CTNNB1, β-catenin; BMP2, Bone Morphogenetic Protein 2; DLX5, Distal-less Homeobox 5; SPP1, Osteopontin; COL1, Collagen type I; TCF7, Transcription Factor 7; LEF1, Lymphoid Enhancer Binding Factor 1; C-MYC, MYC; CCND1, cyclin D; JUN, c-JUN)

For the purpose of evaluation *in vitro*, BMSCs derived from human species’ bone marrow were taken to experiment. In the results of cell proliferation culture, it is indicated that *Chrysosplenetin* enhances osteoblastic differentiation of hBMSCs towards the osteogenic lineage in a non-dose-dependent manner without affecting the viability of hBMSCs at higher dose and longer culture procedure. Moreover, *Chrysosplenetin* increases bone matrix mineralization and activity of osteoblasts as well as the expressions of osteogenic genes. These findings illustrate that *Chrysosplenetin* may be an efficient drug prototype choice for the treatment of osteogenesis deficiency (Fig. 10).

Wnt/β-catenin signaling pathway is the prominent mechanism broadly developed in bone metabolism as the potential therapeutic approach for most of osteolytic bone diseases [6]. Emerging evidences propose that motivation of Wnt/β-catenin evaluates the ability of hBMSCs differentiation and usually is regarded as the major target of multiple explored natural compounds, like *Berberine* [18], *Saikosaponin-A* [19], and *Icariin* [20]. In our research, during osteoblastogenesis, *Chrysosplenetin* is found to trigger the Wnt/β-catenin pathway significantly, involving *CTNNB1*, *TCF7*, *LEF1*, *CCND1*, *JUN*, and *c-MYC*. Particularly, in the addition of *Chrysosplenetin*, TCF7/LEF1 complex in the Wnt/β-catenin pathway further motivates downstream osteogenic genes, such as *RUNX2*, *DLX5*, *SPP1*, *COL1*, and *BGLAP*, in concordance with its positive effect on differentiation of BMSCs towards the osteogenic lineage. Among them, upregulated transcription factor *RUNX2* contributes greatly to the transformation of BMSCs to osteoblasts [21]. Furthermore, marked degradation of phosphorylation β-catenin is induced by *Chrysosplenetin*, and more β-catenin was translocated into the nucleus.

Noggin is a specific homodimeric glycoprotein induced as BMP antagonist [22]. BMPs are a series of secreted cytokines belonging to a family member of transforming growth factor β [23]. Noggin enables to specifically block the struggle of BMP/BMP receptor integration and subsequently suppresses the activity of osteoblasts [24]. That could be an explanation for the upregulation of coding gene of BMP by *Chrysosplenetin* whereas blocking by Noggin. Furthermore, osteogenic differentiation of BMSCs is under the control of cross-talks of Wnt and BMP signaling [25]. Wnt signaling is found to be an upstream activator of transcriptional activities of BMP in osteoblast, but this bioprocess partially relies on BMP/BMP receptor integration in turn, which was blocked by Noggin [25, 26]. Hence, in our study, the effect of *Chrysosplenetin* on Wnt signaling was significantly attenuated by Noggin.

To investigate the DKK1, an inhibitor of Wnt/β-catenin pathway, DKK1 protein was added into the culture medium to counteract with the function Chrysosplenetin [27]. It was indicated that DKK1 performed a similar function on Wnt/β-catenin pathway as Noggin in our research. In the previous study, it is demonstrated that DKK1 can block the expression of Wnt and suppress the recruitment of β-catenin, both of which are able to inhibit the level of BMP2 [28]. Besides, DKK1 is unable to attenuate the stimulatory function of exogenously applied BMP2 ligand [26]. Therefore, unlike Noggin, DKK1 shows no inhibitory effect on the BMP expression [28]. Even though, the positive effect of *Chrysosplenetin* is still suppressed by DKK1 from other approaches.

The *in vivo* evaluation of the function of *Chrysosplenetin* in estrogen deficiency-induced osteoporosis results showed that OVX mice were protected against bone loss by *Chrysosplenetin* treatment via promoting osteoblast formation and function indicated by *Bglap*, with no toxicity *in vivo*. Serum osteoblastic markers including *Ot/Bgp*, *Balp*, and *Ct* were inhibited or positively regulated by *Chrysosplenetin*, which was consistent with the expression of osteoblasts in bone tissue. Hence, it is highlighted that the injected *Chrysosplenetin* promotes the bone formation mainly through serum metabolism.

## Conclusion

Taken together, *Chrysosplenetin* greatly contributes to the osteoblast differentiation through Wnt/β-catenin pathway. This osteogenesis effects induced by *Chrysosplenetin* *in vitro* are revealed to be blocked by DKK1 or suppressed by BMP2 antagonist Noggin. Consistent with the results *in vitro*, these preclinical experimental results implied that *Chrysosplenetin*, as a novel potential and efficient compound, was promising in suppressing estrogen deficiency-induced osteoporosis by promoting the osteoblast differentiation through Wnt/β-catenin pathway.

## Additional file

Additional file 1: PCR reactions used specific primers of the genes. The table provides the information of PCR reactions used specific primers of mRNAs detected in our research, including Runt Related Transcription Factor 2 (*RUNX2*), Osteocalcin (*BGLAP*), β-catenin (*CTNNB1*), Bone Morphogenetic Protein 2 (*BMP2*) were detected. In Strategy two, the mRNAs of osteogenic genes, including *RUNX2*, Distal-less Homeobox 5 (*DLX5*), Osteopontin (*SPP1*), Collagen type I (*COL1*), *BGLAP* and *BMP2*, and Wnt/β-catenin target genes, including *CTNNB1*, Transcription Factor 7 (*TCF7*), Lymphoid Enhancer Binding Factor 1 (*LEF1*), MYC (*C-MYC*), cyclin D (*CCND1*) and c-JUN (*JUN*). (DOCX 15 kb)

## Data Availability

The datasets used and/or analyzed during the current study are available from the corresponding author on reasonable request.

## Abbreviations

ALP: Alkaline phosphatase
BMP2: Bone morphogenetic protein 2
DKK1: Dickkopf-related protein 1
DMSO: Dimethyl sulfoxide
hBMSCs: Human bone marrow stromal cells
OIM: Osteogenic induction medium
OVX: Ovariectomized
PBS: Phosphate-buffered saline
PFA: Paraformaldehyde
