# Supplementary material for: Chrysosplenetin promotes osteoblastogenesis of bone marrow stromal cells via Wnt/β-catenin pathway and enhances osteogenesis in estrogen deficiency-induced bone loss
Source: Stem Cell Res Ther. 2019 Aug 29;10:277. doi: 10.1186/s13287-019-1375-x (PMC6716882; doi:10.1186/s13287-019-1375-x)
Supplement: Supplementary file 1 — PCR reactions used specific primers of the genes. The table provides the information of PCR reactions used specific primers of mRNAs detected in our research, including Runt Related Transcription Factor 2 (RUNX2), Osteocalcin (BGLAP), β-catenin (CTNNB1), Bone Morphogenetic Protein 2 (BMP2) were detected. In Strategy two, the mRNAs of osteogenic genes, including RUNX2, Distal-less Homeobox 5 (DLX5), Osteopontin (SPP1), Collagen type I (COL1), BGLAP and BMP2, and Wnt/β-catenin target genes, including CTNNB1, Transcription Factor 7 (TCF7), Lymphoid Enhancer Binding Factor 1 (LEF1), MYC (C-MYC), cyclin D (CCND1) and c-JUN (JUN). (DOCX 15 kb) [file 13287_2019_1375_MOESM1_ESM.docx]

**Supplemental table,** the primer sequences used in PCR reaction

| **Gene** | **Gene ID** | **Sequence（5’-3’）** | | **Product length （bp）** |
| --- | --- | --- | --- | --- |
| *GAPDH* | NM_001256799.2 | Forward： | CAAGAGCACAAGAGGAAGAGAG | 102 |
|  |  | Reverse： | CTACATGGCAACTGTGAGGAG |  |
| *RUNX2* | NM_001015051.3 | Forward： | GCTTCATTCGCCTCACAAAC | 112 |
|  |  | Reverse： | GTAGTGACCTGCGGAGATTAAC |  |
| *BGLAP* | NM_199173.5 | Forward： | AAATAGCCCTGGCAGATTCC | 105 |
|  |  | Reverse： | CAGCCTCCAGCACTGTTTAT |  |
| *CTNNB1* | NM_001098209.1 | Forward： | CTTCACCTGACAGATCCAAGTC | 98 |
|  |  | Reverse： | CCTTCCATCCCTTCCTGTTTAG |  |
| *BMP2* | NM_001200.3 | Forward： | TGCTTCTTAGACGGACTGCG | 243 |
|  |  | Reverse： | GGGTGGGTCTCTGTTTCAGG |  |
| *DLX5* | NM_005221.5 | Forward： | CCAACCAGCCAGAGAAAGAA | 113 |
|  |  | Reverse： | TAATGCGGCCAGCTGAAA |  |
| *SPP1* | NM_000582.2 | Forward： | CATATGATGGCCGAGGTGATAG | 108 |
|  |  | Reverse： | AGGTGATGTCCTCGTCTGTA |  |
| *COL1* | NM_000088.3 | Forward： | CTAAAGGCGAACCTGGTGAT | 107 |
|  |  | Reverse： | TCCAGGAGCACCAACATTAC |  |
| *TCF7* | NM_001134851.3 | Forward： | GGACAACTACGGGAAGAAGAAG | 133 |
|  |  | Reverse： | TGGGCTAGAGGAAGAAGACA |  |
| *LEF1* | NM_001130713.2 | Forward： | CGGTAACTTGGCTGCATTTG | 98 |
|  |  | Reverse： | GAGGGAACTCTTCCATGAACTC |  |
| *C-MYC* | NM_001354870.1 | Forward： | ATCTCTGGGAGGAATGCTACTA | 95 |
|  |  | Reverse： | ATCTGCGTGGCTACAGATAAG |  |
| *CCND1* | NM_053056.2 | Forward： | GTTCGTGGCCTCTAAGATGAAG | 76 |
|  |  | Reverse： | GATGGAGTTGTCGGTGTAGATG |  |
| *JUN* | NM_002228.3 | Forward： | CCTGATGTACCTGATGCTATGG | 96 |
|  |  | Reverse： | CCTCCTGAAACATCGCACTAT |  |
